# In vitro and in vivo *Anti‐Toxoplasma* activity of *Dracocephalum kotschyi* essential oil

**DOI:** 10.1002/fsn3.2021

**Published:** 2020-11-12

**Authors:** Faham Khamesipour, Seyed Mostafa Razavi, Seyed Hossein Hejazi, Seyed Mustafa Ghanadian

**Affiliations:** ^1^ Department of Pathobiology School of Veterinary Medicine Shiraz University Shiraz Iran; ^2^ Department of Parasitology and Mycology Skin Diseases and Leishmaniasis Research Center School of Medicine Isfahan University of Medical Sciences Isfahan Iran; ^3^ Department of Pharmacognosy Isfahan Pharmaceutical Sciences Research Center Isfahan University of Medical Sciences Isfahan Iran

**Keywords:** anti‐*T. gondii* activity, cytotoxicity, *Dracocephalum kotschyi*, essential oil, *Toxoplasma gondii*

## Abstract

*Toxoplasma gondii* is a zoonotic parasite of worldwide importance, responsible for toxoplasmosis in homeotherms. Although treatment options are readily available, most drugs often cause serious side effects. Extracts of *Dracocephalum kotschyi* (*D. kotschyi*) have shown significant pharmacological activity against various parasites, viruses, and bacteria. In this study, we evaluated the anti‐*T. gondii* activity in vitro and in vivo of *D. kotschyi* essential oil. The thiazolyl blue tetrazolium bromide (MTT) method was used to assess the anti‐*T. gondii* activity and cytotoxicity of the essential oil. The presence of *T. gondii* was observed by Giemsa staining, and the viability was evaluated by the trypan blue staining method. Furthermore, the survival rate of acutely infected mice was evaluated by intraperitoneal injecting of the essential oil (50, 100, and 200 mg kg^−1^ day^−1^) for five days after infection with 2 × 10^4^ tachyzoites. Essential oil, negative, and positive controls that showed the best toxoplasmacidal activity were assayed in triplicate at each concentration. The essential oil exhibited the highest anti‐*Toxoplasma* activity with a half‐maximal inhibitory concentration (IC_50_) of 9.94 ± 0.38 µg, with a selectivity index of 2.463. On Vero cells, the CC_50_ of the oil was 24.49 ± 0.96 µg and exhibited a significant anti‐*Toxoplasma* activity. Moreover, the treatment by essential oil significantly increased the survival rate compared to untreated infected control. In conclusion, the essential oil might be a useful compound, and with more testing, it may be an excellent alternative to standard chemical drugs in the treatment of toxoplasmosis.

## Graphical abstract

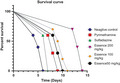

## 1 INTRODUCTION

*Toxoplasma gondii* is a protozoan that causes zoonosis in most homeothermic animals (Khamesipour et al., 2014; McAuley, 2014). The leading cause of infection due to the consumption of water or food contaminated by oocysts of the parasite, excreted by cats. Also, undercooked consumption containing tissue cysts can lead to transmission to humans. Primary infection is usually sub‐clinical. However, in the most severe cases, the manifestations may be eye disease or cervical lymphadenopathy. Maternal infection may also occur during pregnancy. The consequences can be severe for the fetus (Dunay et al., 2018). In animals, this can lead to abortion, weak newborns, or deformities (Weissmann, 2003).

The treatment of toxoplasmosis involves anti‐parasitic drugs that act primarily against the tachyzoite form; they do not eradicate the encysted (bradyzoite) stage. The most effective agent is the pyrimethamine, which is used in most drug regimens (Dunay et al., 2018). However, leucovorin must be given concomitantly to prevent bone marrow suppression. The efficacy of trimethoprim, azithromycin, dapsone, clarithromycin, contrimaxole, atovaquone, and sulfamethoxazole is unclear; therefore, it is recommended that they are used only in combination with pyrimethamine (Dunay et al., 2018; Rajapakse et al., 2013). Of the available combinations, the most effective include pyrimethamine and sulfadiazine or trisulfapyrimidines. However, these combinations have many side effects that hamper their use. Hypersensitivity reactions, megaloblastic anemia, bone marrow damage, and agranulocytosis are inevitable in many cases (De La Torre et al., 2014; Guaraldo et al., 2018). Therefore, it is necessary to find new, less toxic drugs for the treatment of this infection.

Increasingly, natural substances derived from medicinal plants are an essential source of drugs for clinical use. For example, therapeutic properties and active substances to *D. kotschyi* (Heydari et al., 2019). The medicinal plant contains different essential oils compounds, such as citral, α‐pinene, caryophyllene, Gerania, terpinyl, 1,1‐dimethoxydecane, geranial, limonene‐10‐al, acetate, terpinen‐4‐ol, limonene, α‐terpineol, δ‐3‐carene, and others (Heydari et al., 2019; Sadraei et al., 2016), and flavonoids such as xanthomicrol, apigenin 4′‐O‐β‐d‐glucopyranoside, acacetin 7‐O‐β‐d‐glucopyranoside, calycopterin, isokaempferide, luteolin, apigenin, luteolin 7‐O‐β‐d‐glucopyranoside, luteolin 3′‐O‐β‐d‐glucuronide (Fattahi & Nazeri, 2013; Gohari et al., 2003; Saeidnia et al., 2005; Saeidnia et al., 2004; Zeng et al., 2010), limonen‐10‐al, and limonene, among others (Jalaei & Fattahi, 2015; Javidnia et al, 2005). Besides, essential oil from shoots is one of the main active ingredients of *D. kotschyi* (Heydari et al., 2019) and has various pharmacological activities, including anti‐inflammatory (Sadraei et al., 2016) and anticancer activities (Fattahi & Nazeri, 2013).

Although several scientific works have demonstrated the potential medicinal effect of *D. Kotschyi* essential oil on several pathogens, few data are available about its anti‐parasitic activity. Some flavonoid isolated from *D. Kotschyi* essential oil had trypanocidal properties on *Trypanosoma cruzi* epimastigotes (Gohari et al., 2003), but there is, to our knowledge, no data relating to the anti‐Toxoplasma properties of the plant. Given the therapeutic failure and severe side effects of the synthetic drugs available against many pathogens, safer therapeutic options must be explored. Incidentally, the use of essential oils to control parasitic pathogens could be useful in the fight against various infectious diseases. Here, we studied the activity of *D. Kotschyi* essential oil against *T. gondii* tachyzoites.

## 2 MATERIALS AND METHODS

### 2.1 Ethics approval

All applicable international and institutional guidelines for conducting the study. The study protocol was approved by the ethical committee of Shiraz University (IR.Shirazu.REC.1399.1214). The guideline of the Institutional Animal and Ethics Committee, Shiraz University, was used to work in this study.

### 2.2 Plant material

Plant samples were collected from the province of Isfahan, Iran, in October 2019. Authentication was carried out by the Department of Pharmacognosy. A voucher specimen was deposited in the School of Pharmacy herbarium (No. 1519). After drying in the shade, the plant organs were mechanically sprayed using an electric blender.

### 2.3 Preparation of essential oil

The drying of the aerial parts of the plants was carried out at room temperature, in the shade. The essential oils were obtained by hydrodistillation using a Clevenger device for 3 hr. The oil was dissolved in hexane (Merck, Darmstadt, Germany), dried on anhydrous sodium sulfate, and stored at 4–6°C in sealed vials in the dark (Sonboli et al., 2019).

### 2.4 Gas chromatography‐mass spectrometry (GC/MS) analysis

An Agilent 7890A GC coupled with an Agilent 5975C mass detector with triple quadrupole mass analyzer and electronic ionization (EI) was used for the GC analysis of the essential oil. The gas chromatograph was prepared with an HP‐5 GC capillary column (30 m × 0.25 mm; film thickness 0.25 μm). The oven temperature was started from 50 ºC, held for 2 min, raised by 8ºC/min up to 250°C, followed by 250–330°C by 3ºC/min with the total run time of 58 min. The carrier gas was helium was at a flow rate of 2 ml/min. The temperature used for the injector and the detector is 280°C. The parameters for MS were as follows: ion source temperature (230°C), mass range (50–700), ionization voltage (70 Ev). The MSD ChemStation was used as operating software. A comparison of mass spectra and retention times with literature data helped to identify compounds (Javidnia et al., 2005).

### 2.5 Tachyzoites multiplication, quantitation, and maintenance

Tachyzoites of *T. gondii* RH strain were maintained by intraperitoneal passages in Balb/c mice and collected in phosphate‐buffered saline (PBS), pH 7.2, at 3–4 day intervals. Peritoneal fluid was obtained from the infected mice and centrifuged for 10 min at 200 ***g*** at room temperature. This centrifugation removed host cells and debris. Subsequently, the supernatant containing the parasites was collected and centrifuged for 10 min at 1,000 ***g***. The pellet was washed in two stages, first with PBS at pH 7.2 and then RPMI‐1640 (Gibco, USA) without bovine fetal serum.

The viability of the parasites was assessed 30 to 40 min after removal from the peritoneal cavity, using the trypan blue exclusion method. To determine the number of tachyzoites, light microscopy with a hemocytometer was used. The tachyzoites were inoculated into a 75‐ml tissue culture flask containing proliferative Vero cells in a humidified 5% CO_2_ incubator at 37°C (Bajelan et al., 2020).

### 2.6 Animal model

The animal testing work was carried out under international standards. Inbred Balb/c mice were used. The weight of the animals is between 18 and 20 g. The animals were kept in cages under standard laboratory conditions at an average temperature of 20–25°C. During the experiment, the animals had regular access to drinking water and foods.

### 2.7 Vero cells

The kidney cell lines "Vero" were initiated from a green monkey kidney and were obtained from the National Cell Bank of Iran (NCBI, Pasteur Institute of Iran, Tehran, Iran). Vero cells were maintained and cultures at 37°C with 5% CO_2_, in RPMI‐1640 medium supplemented with 100μg/ml streptomycin, 100 units/ml penicillin (Gibco, Pen‐Strep15140), 2 raM l‐glutamine, and 10% fetal bovine serum (FBS) (Bovogen, Australia).

### 2.8 Cytotoxicity assessment

The cytotoxicity of the essential oil was evaluated on Vero cells with a modification of the MTT test (Sigma‐Aldrich, USA) with 3‐(4, 5‐dimethylthiazol‐2‐yl)‐2,5‐diphenyltetrazolium bromide, described by Mosmann (Mosmann, 1983), using 96‐well plates. 6 × 10^4^ cells/mL of Vero cells were inoculated into each well previously containing 100μL growth medium. Incubation at 37ºC followed in an incubator moistened with 5% CO_2_ for 24 hr. After this incubation, the Vero cells were treated with extracts of *D. kotschyi* at different concentrations: 1,000, 500, 100, 50, 10, and 1 μg. Each concentration was added to each respective 96‐well plate. Pyrimethamine and sulfadiazine (reference drugs used in the treatment of toxoplasmosis) were used as positive controls while RPMI‐1640 was used as a control buffer. After 24 hr, the supernatant was removed. 100 μl of the MTT‐PBS solution (5 mg/ml) in RPMI‐1640 in a 1:9 ratios in each well. The plate was then covered with aluminum foil and incubated for 4 hr in a 37º C incubator. The medium was then discarded, and 100 μl of DMSO was added to each well to solubilize the dark blue MTT formazan salt. The optical density was measured at an absorbance of 570 nm using a Dynex microplate reader.

### 2.9 In vitro infection and MTT test

Vero cells were used for this step. The cells were obtained by exponential growth. 3 × 10^5^ parasites/ml was deposited in each well to have a final volume of 200 µl (Belloni et al., 2003). The infected cells were then washed with RPMI‐1640 FBS‐free medium (twice), six hours after inoculation, to remove extracellular parasites. After incubation for 18 hr, 100 μl of RPMI‐1640 medium with 2% FBS were added to each well with different concentrations of essential oil/pyrimethamine and sulfadiazine (to a final concentration of 1‐1000μg) (Sheng‐Xia et al., 2008). The treatment lasted 24 hr, the anti‐*T. gondii* activity and the cytotoxicity of the essential oil were determined by a thiazolyl blue tetrazolium bromide (MTT) method in 96‐well plates (Sigma, St. Louis, MO, USA). The essential oil was added to the cell line. After 24 hr, the MTT solution was added to the cells, and the presence of purple matrices was analyzed using a plate reader. All data points represent the average of three independent experiments. The mean inhibitory concentration (IC_50_) was determined based on the concentration of extracts, controls, and essential oil that successfully inhibited 50% of the *T. gondii* tachyzoites. Selectivity is the mean IC_50_ value for Vero cells relative to the mean IC_50_ value for *T. gondii* (Park et al., 2003).

$$\text{SI}\left(\%\right)=\left(V‐{\text{IC}}_{50}/T‐{\text{IC}}_{50}\right)100$$

T‐IC_50_ and V‐IC_50_ are the median inhibitory concentrations required to inhibit *T. gondii* and Vero cells, respectively.

### 2.10 Tachyzoite viability by trypan blue exclusion

Tachyzoite viability test was permed, in vitro using the method proposed by Cover and Gutteridge (Cover & Gutteridge, 1982). 45 μl of tachyzoite suspension containing 10^6^ cells/ml and *D. kotschyi* essential oil at six different concentrations (1, 10, 50, 100, 500, and 1000µg) in 96‐well microplates are brought together. The whole is incubated at 37°C. After 30, 90, and 180 min incubation in 5% CO_2_ at 37°C, a trypan blue dye exclusion test for tachyzoites is carried out under an inverted microscope. Results were expressed as % viability. Positive controls consisted of 96‐well plates containing pyrimethamine and sulfadiazine at a concentration of 100 mg/ml, while PBS were the negative controls. The plates were then spread on a glass slide followed by an examination under an optical microscope. The experiments were repeated three times.

### 2.11 Light microscopy of tachyzoites in cell lines

Vero cell culture (2 x 10^5^ cells/ml) was carried out on a glass slide in a 35 mm cell culture dish up to the confluence and then infected with 1 × 10^6^ tachyzoites/well. After 4 hr incubation, the monolayers were washed with Hanks' Balanced Saline Solution (HBSS; Gibco Inc., USA), followed by the addition of the essential oil contained in RPMI‐1640. The glass lids were removed from the dishes at 48 hr after adding the essential oil, pyrimethamine, sulphadiazine, and 1% DMSO (negative control). All glass slides were washed with HBSS and fixed with methanol before staining with Giemsa (Sigma Inc., USA). The samples were observed under an oil‐immersion objective lens at a magnification of 1,000 × by an optical microscope. A camera and software were used to capture the images (Sheng‐Xia et al., 2008).

### 2.12 In vivo toxoplasmicidal studies

For in vivo determination of anti‐*Toxoplasma* activity, 60 female Balb/c mice between 4 and 6 weeks of age were used. The animals received an intraperitoneal (IP) injection of *T. gondii* RH (2 × 10^4^ tachyzoites) and then divided into 6 groups of 10 mice (Ebrahimzadeh et al., 2017). After inoculation, batch treatment was immediately started and continued for 5 days at regular 24‐hr intervals as follows: essential oil (200, 100, and 50 mg/kg‐1/day‐1), pyrimethamine (25 mg/kg‐1/day‐1), PBS (negative control), and sulfadiazine (500 mg kg^−1^ day^−1^) (positive control). Regular daily monitoring of the mice was carried out throughout the experiment. Unusual observations and deaths of the mice were recorded. To avoid side effects of the drugs used, the experiment was preceded by a preliminary study in which Balb/c mice were given the same dose of drugs. No clinically significant toxicity or mortality was observed.

### 2.13 Statistical analysis

Statistical analysis was performed using ANOVA using SPSS software (ver 18.0). All values were expressed as the mean ± *SD*, and the level of significance was assumed as *p* < .05.

## 3 RESULTS

### 3.1 GC/MS analysis

**Figure 1:**
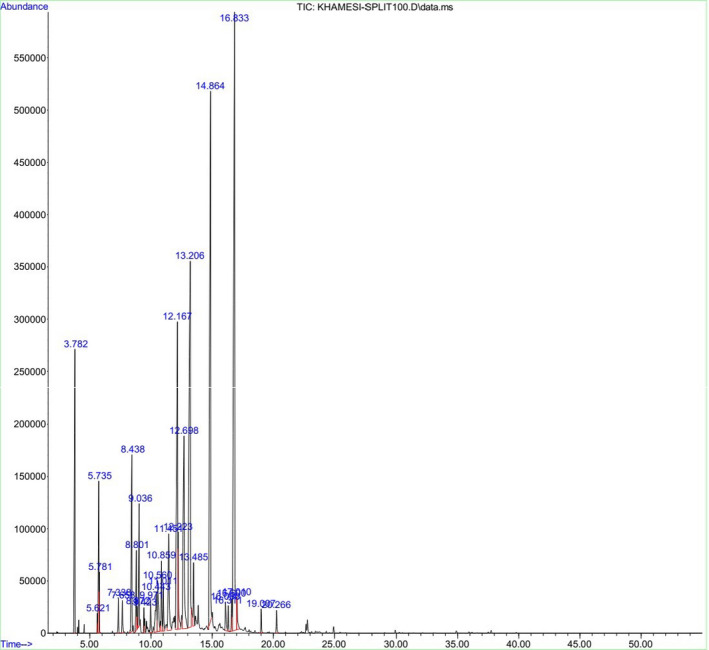
Typical GC‐MS total ion current (TIC) chromatograms

As shown in Table 1, 11 substances were identified. They represent 91.5% of the oil. The main substances were Copaene (22.15%), Methyl geranate (16.31%), Geranial (13.78%), and Carvone (11.34%). Figure 1 presents the typical total ion current chromatograms of the essential oil.

**Table 1:** Chemical composition of the oil of *Dracocephalum kotschyi*

Compound		Molecular formula	RI	Rt (min)	TIC (%)
*1*	*alpha‐Pinene*	C10H16	1,027	3.7597	4.8568
2	l‐Limonene	C10H16	1,090	5.731	3.3306
3	α‐Campholenal	C10H16O	1,126.5	8.4237	3.8666
4	trans‐Verbenol	C10H16O	1,145.91	9.0193	2.8767
5	cis‐Dihydrocarvone	C10H16O	1,196.46	10.5712	2.9758
6	Carveol	C10H16O	1,223.15	11.4436	3.1408
7	Carvone	C10H14O	1,244.52	12.1483	11.3492
8	Chavicol	C9H10O	1,260.55	12.6768	6.8022
9	Geranial	C10H16O	1,275.81	13.1801	13.7829
10	Methyl geranate	C11H18O2	1,326.6	14.841	16.3141
11	Copaene	C15H24	1,387.09	16.804	22.1548
Monoterpenoids: Non‐oxygenated					8.1874
Monoterpenoids: Oxygenated					53.7701
Sesquiterpenoids					22.1548
Phenyl propanoid					6.8022
Minor Compounds less than 1%					8.55

Abbreviations: RI, retention indices; Rt, retention time; TIC, total ion count.

### 3.2 Cytotoxicity activity and in vitro infection and effectors by MTT assay

**Figure 2:**
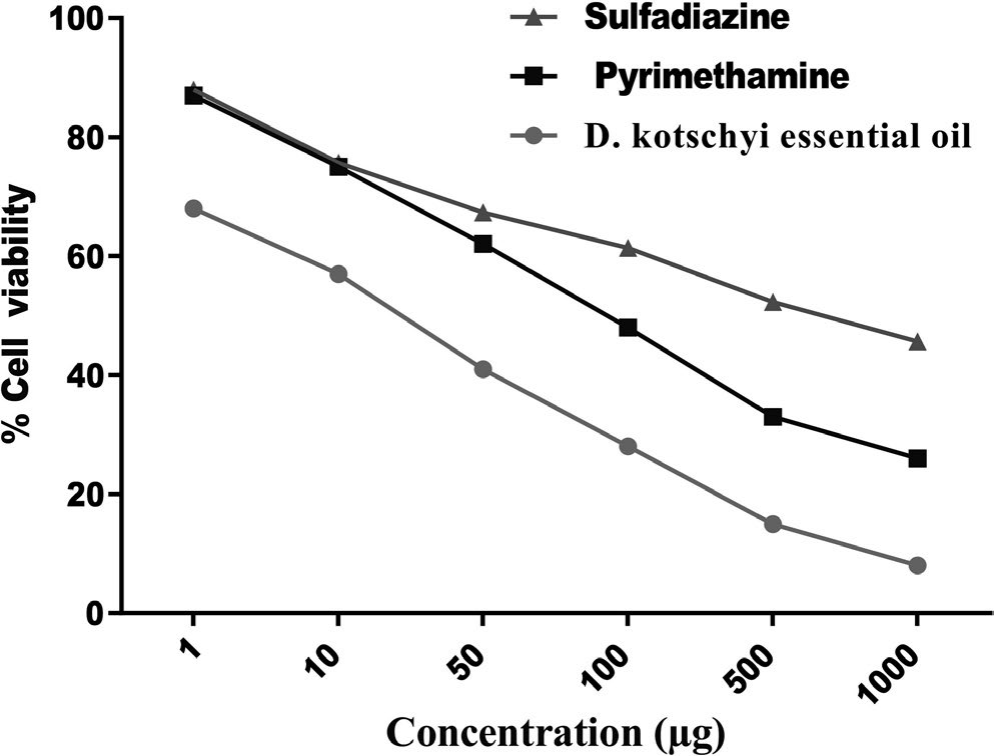
Effects of*D. kotschyi*essential oil on the viability of*T. gondii*tachyzoites

The MTT assay showed that different concentrations of both essential oils of *D. kotschyi* had no cytotoxicity comparatively with the negative control cells (Figure 2 and Table 2). Thus, positive control and different concentrations of *D. kotschyi* were used for further experiments against *T. gondii* in vitro.

**Table 2:** In vitro IC50/ CC50 values for Vero cells (CC50 Vero + essential oil), anti‐*T. gondii* RH strain activity and selectivity of *D. kotschyi* essential oil, sulfadiazine, and pyrimethamine

Tested drugs name	Cytotoxicity (CC50, µg) (Vero + essential oil)	In vitro anti‐*Toxoplasma* assay (IC50, µg)	Selectivity index (SI)^a^
Essential oil	24.49 ± 0.96 µg	9.94 ± 0.38 μg	2.463
Sulfadiazine (Positive control)	607.18 ± 0.89 μg	391.18 ± 0.97 μg	1.552
Pyrimethamine (Positive control)	342.23 ± 0.99 μg	84.20 ± 0.93 μg	4.064

Note

IC_50_, median inhibitory concentration.

^a^ Ratio of the CC_50_ value for Vero cells to the IC50 value for *Toxoplasma gondii* tachyzoites.

Table 2 and Figure 2 presents the results of in vitro anti‐*T. gondii* activity and selectivity. The essential oil exhibited the highest toxicity against *T. gondii* tachyzoites at 1,000 μg (Figure 2). The in vitro cytotoxicity assay indicated that the essential oil compounds had toxicity on the growth of *T. gondii* tachyzoites at concentrations as lower than 9.94 ± 0.38μg with a selectivity index (SI) of 2.463. Cytotoxicity (CC50, μg) (Vero + essential oil) produced better activity against the cell line. The essential oil was active against *T. gondii* tachyzoites with an IC50 value below 10 µg (Table 2).

### 3.3 Effects of essential oil on tachyzoites viability

Six concentrations (1, 10, 50, 100, 500, and 1,000 µg) of *D. kotschyi* essential oil were incubated for 30, 90, and 180 min. The trypan blue stain was used to assess the viability of the tachyzoites. The results showed that *D. kotschyi* has acceptable efficacy in vitro, and the parasiticidal effect of essential oil was significantly better than positive control in all exposure times (*p* < .05) (Table 3).

**Table 3:** Effects of essential oil on tachyzoite viability

Groups	Concentrations μg	Time			*p*‐Value
		30 min	90 min	180 min	
Essential oil	1	96.33 ± 1.52	90.66 ± 1.15	86.66 ± 1.52	*p* < .05
	10	95.00 ± 1.00	86.33 ± 1.52	82.66 ± 2.08	
	50	90.66 ± 1.52	86.66 ± 1.52	80.00 ± 2.00	
	100	90.33 ± 1.15	81.00 ± 1.00	78.33 ± 2.51	
	500	87.33 ± 1.15	77.00 ± 2.00	70.66 ± 2.51	
	1,000	79.66 ± 1.15	72.66 ± 2.08	63.66 ± 2.51	
Sulfadiazine (Positive control)	1	97.66 ± 0. 57	92.33 ± 1.52	90.66 ± 1.52	*p* < .05
	10	97.33 ± 0.57	96.00 ± 1.00	90.00 ± 1.00	
	50	95.66 ± 1.52	94.66 ± 0.57	88.66 ± 1.15	
	100	95.00 ± 2.00	89.66 ± 1.52	87.66 ± 1.52	
	500	93.66 ± 1.52	87.33 ± 2.08	85.66 ± 2.08	
	1,000	90.33 ± 2.08	84.66 ± 1.52	79.33 ± 1.52	
Pyrimethamine (Positive control)	1	95.66 ± 1.52	92.00 ± 2.00	84.66 ± 2.51	*p* < .05
	10	93.33 ± 2.08	87.33 ± 2.08	81.66 ± 2.08	
	50	93.33 ± 3.05	84.33 ± 3.51	77.33 ± 2.51	
	100	92.00 ± 2.00	84.33 ± 1.52	76.66 ± 1.52	
	500	90.00 ± 2.00	80.33 ± 2.51	75.33 ± 2.51	
	1,000	86.33 ± 1.52	75.33 ± 2.08	73.00 ± 3.00	
Negative	‐	97.00 ± 1.00	95.33 ± 2.51	95.33 ± 1.15	*p* > .05

This essential oil at the concentration of 1 µg killed 96.33, 90.66, and 86.66% of tachyzoites after 30, 90, and 180 min, respectively. The concentration of 1,000 µg died 79.66, 72.66, and 63.66% of tachyzoites after 30, 90, and 180 min, respectively.

The effect of *D. kotschyi* essential oil against *Toxoplasma* was highly significant compared to the negative control (*p* < .05). Besides, a significant difference was found between the essential oil of *D. kotschyi* and the positive control after different exposure times and at all concentrations.

### 3.4 Light microscopy of morphology tachyzoites in Vero cell lines

**Figure 3:**
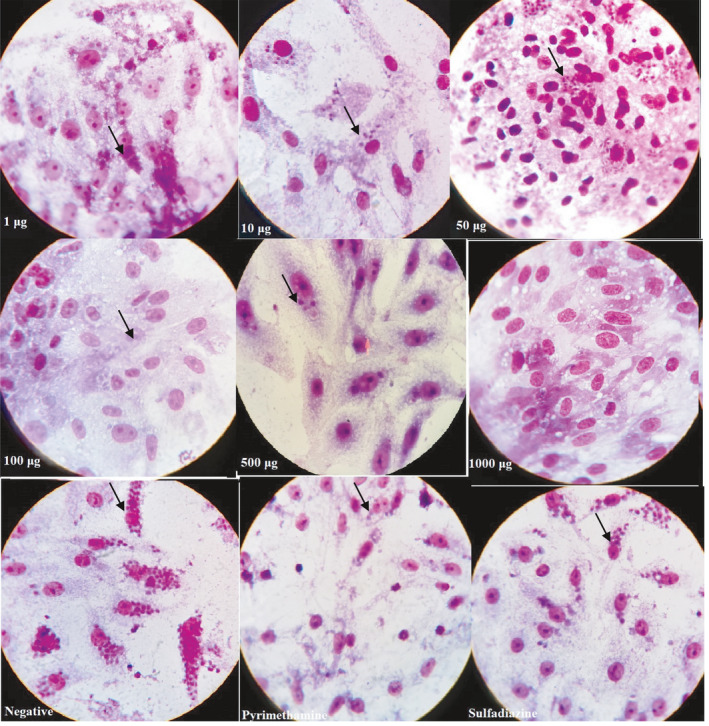
Light microscopy images of Vero cells, stained with modified Giemsa, are shown in different groups. The regular rosette arrangement of the intracellular tachyzoite, as seen in the negative control, was lost in the group treated with*D. kotschyi*essential oil. The tachyzoites were shown with arrows.

The effects of increasing concentrations of *D. kotschyi* essential oil treatment on *T. gondii* in Vero cells were investigated by light microscopy (Figure 3). It shows the morphological effects of RPMI‐1640, positive control, and essential oil of *D. kotschyi* on Vero cell and *T. gondii*‐inoculated Vero cells. The number of tachyzoites decreased sharply from 1 µg to 1,000 µg of essential oil compared to the positive and negative control. The test was performed on tachyzoites from peritoneal exudates using Giemsa staining. Considerable numbers of the collected tachyzoites from all treated subgroups were reduced (Figure 3). Microscopic observation of intracellular parasite postinfection treatment was performed. Treatment of infected cells with *D. kotschyi* essential oil, treatment with the positive control, and negative control are shown in Figure 3. Vero cells showed no remarkable morphological changes.

### 3.5 In vivo toxoplasmicidal studies and survival rate

**Figure 4:**
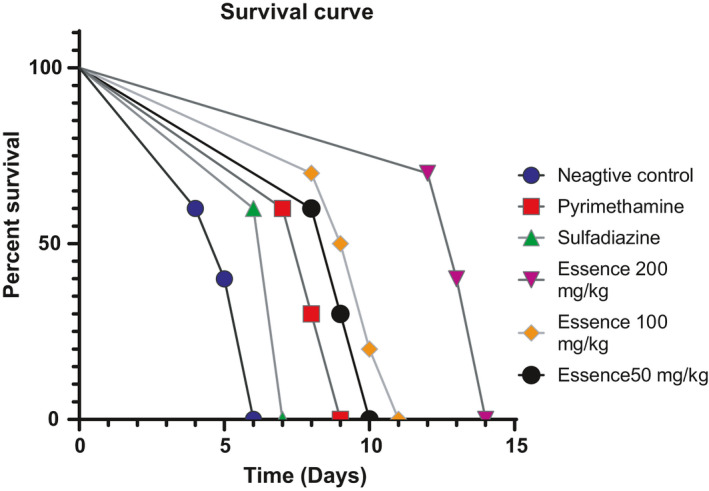
The survival curves of mice following acute toxoplasmosis. Balb/c mice infected with*T. gondii*RH strain were treated with*D. kotschyi*essential oil, Sulfadiazine, and Pyrimethamine

The mice were observed daily, with observations recorded 14 days after treatment. From the fourth‐day postinfection, progressive mortality was recorded in the infected, untreated groups. By the sixth day, all mice were carried in this batch. In the group of infected mice. Mice treated with 50, 100, and 200 mg kg^−1^ day^−1^ of *D. kotschyi* essential oil, the mice started to die on the 8th, 8th, and 12th day after treatment, respectively. Mice of sulfadiazine groups (positive group) began to die on the sixth day until the seventh day. Mice of pyrimethamine groups started to die on the seventh day until the ninth day, and the mice of *D. kotschyi* essential oil groups began to die on the eighth day until the fourteenth day. A significant difference in the survival time between all treatment subgroups was observed. The treatment with *D. kotschyi* essential oil (200 mg kg^−1^ day^−1^) leads to better results in mice survival than treatment with *D. kotschyi* essential oil (50 and 100 mg kg^−1^ day^−1^). Mice in the treatment groups of *D. kotschyi* essential oil (50, 100, and 200 mg kg^−1^ day^−1^) showed a statistically higher survival rate compared to untreated infected control (*p* < .05). There was a significant difference between *D. kotschyi* essential oil (50, 100, and 200 mg kg^−1^ day^−1^) groups with positive control (*p* < .05) (Figure 4).

## 4 DISCUSSION

Studies to explore alternative treatments for toxoplasmosis are limited, particularly those involving medicinal plants. In the sphere of natural substance enhancement against toxoplasmosis, there are no data on the essential oils of *D. kotschyi*. In this study, we evaluated the effect of treatment with *D. kotschyi* on *T. gondii* infection in vivo and in vitro. Various studies have evaluated the anti‐*Toxoplasma* effects of many herbal extracts in vitro and in vivo, but research on relatively useful and low toxicity substances is still needed (Al Nasr et al., 2016; Arab‐Mazar et al., 2017; Montazeri et al., 2017; Sharif et al., 2016).

The essential oil of *D. kotschyi* had potent anti‐*T. gondii* activity. It exhibited cytotoxic effects against many cell lines tested and showed a more significant preferential cytotoxic effect of *T. gondii* tachyzoites, on all cell lines tested. Effective treatment for *T. gondii* has remained unavailable for decodes even as new drugs continue to enter the market. These data are of interest, particularly with a view to the development of new therapies (Konstantinovic et al., 2019).

The essential oil had an inhibitory action on the multiplication of the parasite with an IC_50_ of 9.94 µg μg/ml. The reduction in the number of *T. gondii* tachyzoites was concentration‐dependent. In a previous study, the anti‐*T. gondii* activity of nutmeg essential oil was demonstrated with an EC_50_ value of 24.49 µg μg/ml. Other studies have shown that the extract of *D. kotschyi* had antimicrobial activities but also cytotoxicity toward cancer cells. Furthermore, according to these earlier studies, essential oils of *D. kotschyi* were more potent against Gram‐positive bacteria (Orlanda & Nascimento, 2015). The essential plant oil was thought to have effects on parasites such as *T. gondii* tachyzoites, and this was proved in the current study. Although further tests on its efficacy at large scales are necessary, the present study shows the potential of *D. kotschy* essential oil as an alternative treatment. These in vitro pharmacological data attribute to the essential oil a potent anti‐*T. gondii* activity coupled with an absence of toxicity to the host. There are no previous published studies on the effect of the essential oil against the RH strain of *T. gondii* in a mouse model. In this study, the mouse model, we found that essential oil significantly reduced the parasite number.

Cytotoxicity assays showed that *D. kotschy* was not cytotoxic to Vero cells. This result is consistent with that obtained in a previous study (Sharif et al., 2019) with ethanolic extract of *Tinospora crispa*. Furthermore, *D. kotschyi* protected mice with acute toxoplasmosis from death to a certain extent and increased the survival in mice in vivo. In a study conducted by Pillai et al., the essential oil of Nutmeg (*Myristica fragrans* Houtt) showed strong anti‐*T. gondii* activity beside low toxicity against a normal cell line (Pillai et al., 2012). Our results showed that *D. kotschyi* is active against tachyzoites of *T. gondii* in vitro, and the tachizoitocidal effects of essential oil were significantly better than control. There was a significant difference between essential oil of *D. kotschyi* and control groups in all exposure times.

So far, several studies have been carried out on the therapeutic potential of natural products against *T. gondii* (Kavitha et al., 2012; Devanthran et al., 2017; Ahmadpour et al., 2019). For example, Devanthran et al. (2017) showed significant anti‐*Toxoplasma* activity of *Piper sarmentosum* ethanol extract (IC_50_ = 12.4 μg/mL). Also, after 24 hr of exposure to the extract, inoculated Vero cells exhibited low parasitemia associated with no specific morphological changes (Devanthran et al., 2017). As for Ahmadpour et al. (2019), they demonstrated the highly significant anti‐*Toxoplasma* effect of methanolic extracts of *Zea mays* and *Eryngium caucasicum*. *Z. mays* (10, 25, and 50 mg/ml) killed 100% of the parasites after 180 and 120 min of exposure, respectively. Besides, the treatment of mice experimentally infected with *Z. mays* (100, 200 mg kg^−1^ day^−1^) and *E. caucasicum* (100 mg kg^−1^ day^−1^) significantly increased their survival rate compared to untreated infected mice (Ahmadpour et al., 2019). All these results agree with the findings of the current study.

Another study by Moine et al. showed strong in vitro activity of eight compounds with a biphenylimidazoazine scaffold against *T. gondii*, with a 50% effective concentration (EC_50_) below 1 mM, coupled with an absence of toxicity on human fibroblast cells. However, in vivo, investigations are necessary to confirm the activity of these compounds (Moine et al., 2015).

Evaluation of anti‐*T. gondii* effects of *D. kotschyi* in mice acutely infected by the RH strain of *T. gondii* had survived at 14 days post‐treatment. Our study showed that the essential oil of *D. kotschyi* could inhibit *T. gondii* in mice. These results are consistent with other studies (Choi et al., 2013; Kareshk et al., 2015; Leesombun et al., 2016; de Oliveira et al., 2009). Leesombun et al. (2016) reported that *Thai piperaceae* extracts inhibited the proliferation of *T. gondii* tachyzoites (Leesombun et al., 2016). Also, Kareshk et al. (2015) showed that the essential oil of *Bunium persicum* (0.1 ml/kg) reduced significantly mean several parasites compared to the control one (Kareshk et al., 2015). Moreover, the results by Choi et al., showed that *Zingiber offcinale* (Ginger) extract courses improved the survival of infected mice and also inhibited the inflammatory response (Choi et al., 2013).

Choi *et al*. reported that, 3‐[{2‐((E)‐furan‐2‐ylmethylene) hydrazinyl}methylene]‐1,3‐dihydroindol‐2‐one (ATT‐5126) and 6‐trifluoromethyl‐2‐thiouracil (KH‐0562) showed higher selectivity index compared with Pyrimethamine (PYR) in treatment of toxoplasmosis (Choi et al., 2014). Also, Zhang *et al*. indicated that SI of Martine and Oxymartine parasite were higher compared with spiramycin in HeLa cells (Zhang et al., 2016). The present study demonstrates that the essential oil of *D. kotschyi* contains compounds possessing anti‐parasite properties. The presence of copaene, methyl geranate, geranial, carvone, 3‐carene, alpha‐pinene, limonene, and carveol compounds in these plants may be responsible for their anti‐parasitic effect on *T. gondii* infection. Traditionally, the plant has long been used for therapeutic purposes (Heydari et al., 2019).

The activity of the essential oil is due to the quality of the active molecules present in the essential oil. The previous study reported the presence of active compounds in the plant extract or oil. Shakib et al. (2018) found that *D. kotschyi* essential oils contain Oxygenated Sesquiterpenes, Monoterpene Hydrocarbons, Sesquiterpene Hydrocarbons, and Oxygenated Monoterpenes. Flavoinoids such as calycopterin, acacetin 7‐O‐β‐d‐glucopyranoside, xanthomicrol, apigenin 4′‐O‐β‐d‐glucopyranoside, isokaempferide, luteolin 3′‐O‐β‐d‐glucuronide, luteolin, luteolin 7‐O‐β‐d‐glucopyranoside, apigenin have also been identified (Fattahi & Nazeri, 2013; Saeidnia et al., 2004, 2005; Zeng et al., 2010).

Our results further suggest that essential oil treatment may also induce dose‐dependent decreases in the ATP levels in *T. gondii*. This suggests that the mechanism of action of the essential oil against *T. gondii* could involve interference with the mitochondrial function of the parasite (Zhou et al., 2019). Besides, *T. gondii* tachyzoites have only one mitochondrion, unlike mammalian cells (Melo et al., 2000); therefore, mitochondrial damage can disrupt energy metabolism by inhibiting the supply of energy in *T. gondii* tachyzoites, leading to their death. Confirming this, anti‐*Toxoplasma gondii*, under a microscope, the number of tachyzoites showed a sharp decrease from 1μg to 1,000 µg compared to control. However, the specific mechanism of action needs to be further investigated.

## 5 CONCLUSIONS

We report for the first time anti‐*Toxoplasma* activity of *Dracocephalum kotschyi*, in vitro, and in vivo. The results obtained in this study attribute to the essential oil of *D. kotschyi*, inhibitory activity against *T. gondii*, without toxicity to the host. It can be assumed that the mechanism of action of the essential oil against *T. gondii* is associated with the mitochondrial function of *T. gondii*. The essential oil of *D. kotschyi* has an interesting pharmacological potential to be valued in the fight against toxoplasmosis. Supplementary works are necessary to identify actives compounds associated with anti‐*Toxoplasma activity*.

## CONFLICT OF INTEREST

The authors declare that they do not have any conflict of interest.
